# Early Care of Children’s Teeth, with Special Reference to the Prevention of Facial and Oral Deformities

**Published:** 1909-08-15

**Authors:** Milton T. Watson


### EARLY CARE OF CHILDREN'S TEETH, WITH SPECIAL REFERENCE TO THE PREVENTION OF FACIAL AND ORAL DEFORMITIES.

MILTON T. WATSON, D. D. S.

Read before the Michigan First District Dental Society, May 13, 1909.

The teeth and the bony framework designed for their support are merely essential parts of a great complex apparatus, the functions and growth of which are disturbed just in proportion to the importance of the particular part or parts which may be out of order. It so happens that the eruption of the teeth and the development of the jaws plays an important role in the performance of at least two of the most important functions of this great human apparatus, and it therefore becomes a matter of wisdom for us to do everything in our power to prevent any abnormality in this region, for it is especially entrusted to the care of the dentist and the orthodontist. At the same time we must recognize our limitations and call to our aid the rhinologist or the family physician, more especially the former, when their assistance is needed.

The two important factors above referred to are efficient, hygienic masticating apparatus for the proper preparation of our foods, and the development of a full sized dental arch and jaw, so that there may be a fully developed internal face and a normal breathing capacity. If we could completely separate the two—the latter would possibly be the more important, but it so happens that this highly important function—normal respiration—is usually present only in cases where reasonably well developed jaws are found.

Those of you who recently heard that magnificent lecture by Doctor Frederick B. Noyes, must necessarily have awakened to a much keener appreciation of the important role the eruption of the teeth plays in the development of the jaws, and for that matter the whole internal face, and, of course, the lines of the external face are in a large measure dependent upon the development of its internal or bony framework. In view of the highly important connection existing between the eruption of the teeth and the development of the maxilla and mandible, it must be clear to you that the deciduous teeth are entitled to a very much higher type of dental attention than they have ordinarily received. It is not sufficient to simply insert oxy-phosphate cement fillings in approximal cavities in these teeth, for as we all know its gradual dissolution begins almost immediately and the pushing forward of the teeth and process surrounding them, in order that sufficient space may be provided for the eruption of permanent teeth further back in the mouth, is interfered with. The crowding together of these teeth, as the surface of the cement filling is washed away, makes it only a matter of a few months when these deciduous teeth occupy considerably less space mesio-distally than was originally intended. This calamity, for such it really is, has to be reckoned with later on when the eruption of the permanent teeth follows the loss of these particular deciduous teeth. There is, to my mind at least, no doubt but that from a health standpoint, it would be better and cheaper for those most vitally concerned, if the deciduous teeth were always filled with materials of as nearly an indestructible character as possible. It is quite as important, possibly even more important, that these fillings should be fully contoured, as it is that this particular part of the work should receive careful attention in the operations you do upon permanent teeth.

There is another highly important function belonging to the deciduous dentition, namely, that of mastication and by this means thorough insalivation of the food. There is no time in life when good digestion and proper assimilation is of more importance than during the period of stress through which a growing child passes, and it is especially true that there is no other time in life when the masticating capacity is, of necessity, more seriously impaired, because of the time that must elapse between the loss of the deciduous tooth and the full eruption of its permanent successor. If the temporary teeth are allowed to go without proper dental attention they soon become sensitive to thermal changes, to sweet and sour things and eventually tender to the touch, so that the inclination to masticate is avoided and this, of course, means that the food enters the stomach without being properly prepared for digestion, and the stomach will not long continue to do double duty without some attendant evil developing.

There is still another phase of the care of deciduous teeth which deserves our serious consideration. I refer especially to the cases where these teeth have been allowed to get into a very bad condition before the family dentist is consulted. This would not happen if parents could be made to realize what a tremendous amount of the child's vital force is consumed in overcoming and destroying the poisonous and fnfected material which is constantly swallowed where there are large cavities in the teeth filling with decomposing food stuffs. This stage is, of course, followed later by the development of alveolar abscesses and the swallowing of really large quantities of pus. Where abscesses exist it is not enough that you should open them and relieve the acute suffering, but they should be treated, cured and the teeth filled with the care their importance fully entitles them to. Where a deciduous tooth has gone beyond repair and the comfort of the child demands its removal, the space thus produced should invariably be retained mechanically unless the permanent tooth can reasonably be expected to erupt within a *very few months*. I have repeatedly seen children whose deciduous molars had been lost permaturely, suffer from a marked additional loss of masticating capacity by having all four first permanent molars rendered practically useless because they had tipped forward into the spaces formerly occupied by the deciduous molars. This tipping, together with more or less rotation of these molars, renders them of little use for masticating purposes, and at the same time interferes with the development of the jaws, and allows these teeth to encroach upon a space that is already insufficient for the bicuspids and cuspids.

I have advanced nothing new nor very radical regarding the importance of the teeth at this particular period of human existence, neither have I advanced any new theories in regard to the method of caring for the teeth, but I have, on the other hand, *endorsed* methods which, while they are not new, are, nevertheless, not in common practice. When the profession fully awakens to the truth and is better prepared to intelligently instruct the layman, he will not only much more willingly, but gladly expend the additional fee, which will be demanded for work of the character I have referred to, for he will then realize that by so doing he is eliminating to a considerable degree, the probability of marked malocclusion of the permanent teeth with the attendant annoyance and expense of a long period of orthodontic treatment; and more important than this, he will be doing what he can to promote the health and appearance of his child. The child will at the same time be saved the agonies which would otherwise result from carieous and abscessed teeth, and which many of us, who did not receive such attention, can still remember as keenly as we do any of the events of our childhood.

It seems to me that it would perhaps not only be unnecessary but somewhat out of place for me to attempt to discuss with you at any length the materials best suited for restorations in broken down deciduous teeth. As a matter of fact I believe it to be of comparatively little importance whether these fillings be of gold, porcelain, alloy or silver inlays, if such a thing could be made. It is simply important that the material be as nearly indestructible as possible, and one which will enable you to produce proper forms, not only in mesial and distal contours, but in the proper shape of the occluding surfaces as well. Alloy at the present time seems to offer the greatest number of advantages—especially where economy needs to be considered.

It must be remembered that in working toward an ideal we will not receive the encouragement of a majority of the people with whom we come in contact, until that method or ideal has at least grown hoary with age. This should occasion us neither surprise nor disappointment, for we have but to stop and think how slow those of us who are devoting our lives to this particular branch of work are in really accepting modern dental truths. I can easily imagine that there are many men in this audience to-night, who, if they were to express their inmost thoughts, would unqualifiedly condemn the chief recommendations of this paper as being impracticable because of the very fixed notions entertained by laymen in regard to the non-importance of the temporary teeth. There is, of course, an element of truth in this, but on the other hand if the dentist himself were keenly awake to the truth he would readily convince many of his patients of the wisdom of giving the deciduous teeth the very best possible dental attention. This opinion is not expressed merely in a dogmatic sort of way, but is based upon the very fact that it has been my experience repeatedly to see these same people who have either declined or neglected to give their children proper attention, submit to all sorts of inconvenience both financially and otherwise, to have resulting malocclusions corrected. It seems to me then a logical conclusion that they would go to the comparatively little trouble and expense of proper dental attention if the matter were wisely presented to them, when they will go to so much trouble to correct later disturbances, when they finally learn of the associated evils of malocclusion of the teeth.

At the risk of offending some of you I am going to tell you a truth which will strike so near home in many cases that it will almost make you move uneasily in your chair. That painful truth is simply this—that it is in a large measure only the busiest men in our profession who find time to devote to the minute details necessary to do such work as I have outlined. If it is not assuming too much I would like, with only the kindest of intentions, to urge the young men, upon whose hands time drags heavily, to spend some of this time in bringing their work up to a much higher standard of perfection. It will go a long way towards developing an ideal, and also a degree of ability which will not long remain unrecognized or unrewarded.

I have thus far discussed means and methods of repairing injuries that have already occurred, but let me assure you that I believe the time is not far distant when our best energies will be spent in attempting to prevent disease and caries by proper prophylactic measures. I have been greatly pleased and impressed by numerous cases that have fallen under my observation, where little children are receiving judicious prophylactic care and the results amply justify the expenditure of both the time and money involved.

The circle of influences which play a more or less important part in the production of oral and facial deformities is so large that as I have said before, we cannot hope to control them completely by dental attention alone. Let us for a moment stop and consider that malocclusion is the rule rather than the exception, and the enormity of our task in attempting to overcome this condition will begin to dawn upon us. However, I want to discuss with you for a few moments some of the more important of these influences without any attempt at taking them up in the order of their importance, for to the best of our knowledge and belief the predominating influence varies in different cases. I have already devoted considerable time to the discussion of what I believe to be proper dental attention, and I have dwelt upon this at some length because it is the one factor which you as general practitioners will be most concerned with, and also because if ideal conditions can here be established a wide range of beneficial influences will result.

Rhinologists and orthodontists are agreed that masticating exercise is essential to the proper growth and development of the jaws. This being true you will readily see the wisdom of keeping the teeth, both deciduous and permanent, in the best possible condition, for if they are sensitive, mastication is, of course, avoided. It is also well to remember that if the habit of bolting the food is once acquired, that it is one difficult to overcome.

Malnutrition is a condition which naturally comes to our minds when we are discussing mastication for, without proper mastication, the digestion is disturbed, and faulty assimilation must ever accompany faulty digestion. We should also remember that malnutrition in relation to facial and oral deformities is not necessarily a local condition, for it may and often does effect the entire physical economy.

The relation of disturbances of the upper respiratory tract to facial and oral development is one which has been discussed before this society so often that I shall touch upon it lightly. You must remember, however, that any pathological condition which encroaches upon the nasal spaces will interfere with the function of nasal respiration, and that this particular function or exercise is absolutely essential to the development of this part of the internal face. It is also true that anything that disturbs the development of this region, reacts unfavorably upon the development of all adjacent parts. In the more aggravated cases this influence is really of a startling nature, as it has an effect not only upon the development of the local parts, but upon the development of the entire being. In the way of local effects we see in addition to the various irregularities of the teeth, distorted faces, impaired vision and at times even a mental disturbance due to failure of development in the base of the brain case. The general physical disturbances are those which naturally follow when a child receives an insufficient amount of oxygen, especially noticeable in the forms of anemia, various marked nervous disturbances and in a brain fag, which is nearly always noticeable to a greater or less degree in any child suffering from nasal occlusion.

A great many rhinologists have already reached the point where they recognize their inability to cope with nasal occlusion where it has gone on far enough to involve the development of the maxilla to a pronounced degree. It is especially in conditions of this sort where the rhinologists have been called upon to operate three or four times for recurrent adenoids. Such conditions as this can only be handled successfully by the united efforts of the rhinologist and orthodontist, and I am strongly of the opinion that the rhinologist should render his services first so that when the patient comes under orthodontic treatment, he may be able to utilize whatever nasal space he has, for this exercise will in itself aid materially in developing the parts which are already being stimulated through pressure upon the teeth. It is equally true that the services of the orthodontist will avail little unless all marked pathological growths are removed from the nasal and postnasal regions, for it is not possible I think, to develop the maxilla and mandible fully in patients who are habitual mouth breathers.

The effect of hereditary influences is still a disputed question, though it is perhaps one which the majority of orthodontists place in a secondary position. Heredity is, however, a subject upon which an entire evening could be devoted, therefore, any hasty discussion of the subject can avail nothing. I cannot drop the matter, however, without saying I am not in sympathy with the opinion held by the majority of orthodontists, for I believe that heredity together with environment plays a very important role in the etiology of malocclusion of the teeth and the facial deformities which must necessarily accompany malocclusion.

The point which I have been gradually leading up to is this—that regardless of whether the various etiological factors are present singly or in groups, that it is only by mechanical stimulation that sufficient development can be produced to allow a normal arrangement of the teeth, and a normal facial development. I also want to impress upon you as clearly as I can the advisability of early attention in these cases where mechanical stimulation is required. The advantages to be gained by early operation are that you have less to accomplish in order to bring the development up to normal, consequently less physical force will be required and for a shorter time. If perfectly normal conditions can be established early in child life, the probability of normal development occurring from that time on is very good. This, of course, means the elimination of all the various handicapping influences which would otherwise impede the child's development. To put it otherwise, the advantages to be gained by the early correction of malocclusion are that you avoid a continuation of impaired masticating capacity, of interference through abnormal breathing with the development of the jaws and face, for in so far as you are able to improve the breathing capacity you relieve the various conditions associated with mouth breathing, and then you enable the natural forces to produce developmental activity instead of trying at a later time to produce artificially an enormous amount of development which should already have taken place. There is in this connection, however, some misapprehension, not only on the part of the laymen but the dental profession as well. If you will recall the fact that the calcification of the germs of the permanent teeth begins as early as the twenty-fifth week of embryonic life, and that by five years of age the incisal and masticating ends of all the teeth except the third molars have begun to calcify, you will readily understand that anything that interferes with the development of the internal face, even up to the tender age of five or six years, may mean that these developing teeth have been deflected from their normal position, and that they will erupt in rotated positions even though the jaw is developed by artificial stimulation, to a perfectly normal size. Thus you see it is not possible by early attention to prevent entirely the malposition of teeth that will erupt several years later. If the true function of an orthodontist was that of being a beauty specialist, as was at one time believed, then there would be much less gained by very early correction of malocclusion. In view of the fact, however, that malocclusion is now looked upon as merely a symptom of a greater disturbance and that its correction is demanded chiefly from a health standpoint, you will readily see that the burden of evidence is overwhelmingly in favor of early corrections. The careful observation of clinical results together with an earnest study which has been enriched many times by consultations with rhinologists, has forced the conclusion upon orthodontists and rhinologists as well, that where restricted growth is in evidence that the one most important thing to do is to proceed at once to stimulate a renewal of developmental activity.

I might go on at much greater length in this discussion but if you will only put aside your personal prejudices and honestly endeavor to work along the lines suggested for a little while, I am confident that you will become convinced of the truth and will require no further proof. I also remember that on two or three occasions I have transgressed all propriety and imposed myself upon this society for nearly two hours at a time. You were charitable on those occasions, but I realize there must be a limit—hence my discretion to-night.

### DISCUSSION.

Dr. A. A. Laupe. Dr. Watson has shown us this evening that the development of the internal nose and normal breathing capacity is dependent on an efficient masticating apparatus. I think that it must be apparent to all of us that the only logical conclusions that can be drawn from the facts presented here are that there is a close relationship between the development of the teeth and jaws and tissues of the nasal region, and that the greatest amount of good must come from early treatment, whether dental or orthodontic.

The importance of normal mastication and breathing in reference to malnutrition is worthy of serious thought. It must be clear to all that where there is malnutrition the entire physical being must suffer in consequence. Improper mastication must produce improper development, and anything which impairs mastication must also interfere with the proper functions. As mastication is for the purpose of properly preparing the food, this use would be seriously impaired during the exfoliation of the deciduous teeth, if nature had not wisely permitted the first permanent molar to erupt before this time. Therefore, nature must have anticipated this and so caused the first permanent molar to erupt previous to the exfoliation of the deciduous teeth, for the purpose of assuming this important function of mastication. Therefore, I do not agree with the essayist that mastication is seriously impaired during this period if the deciduous teeth are in normal condition.

Children's teeth are for a purpose, the same as the teeth of the adult. If children's teeth are diseased, they are deserving of as thorough and conscientious treatment as the teeth of the adult. A physician does not neglect treating a child just because it is a child; neither should we neglect treating the deciduous teeth just because they are deciduous teeth. It is true that physicians do not administer to children the same form of treatment; neither do dentists agree as to the same kind of treatment for the deciduous teeth. However, the nearer the treatment is to the ideal, the nearer to the ideal will be the results.

To serve little children is not so easy a task. The little child is not an ideal patient, and ideal services cannot always be rendered. Where it is possible to serve with the ideal, the ideal is the best, the easiest, and the only service to render. Where this is not possible, then the ideal must be set aside, and the conditions present in the individual case will necessarily govern the treatment. Thus, even a general rule must be subservient to the rule of the individual. As temporary fillings are destructible fillings, so the best fillings must be indestructable ones. This will not only make a very efficient occluding surface, but will also tend to preserve the proper space for the succeeding teeth; we therefore must agree with the essayist that the best fillings to insert are the permanent ones. It is my opinion that many permanent fillings of cement and amalgam could be inserted where cement is used alone. There may be instances, however, in which it will be best to insert only a temporary filling, or even resort to operative treatment alone, than to attempt to render the ideal and cause untold future damage by making it impossible for any one to perform any dental operations on that child again.

I do not believe that the laity or profession in general will ever agree with the essayist in that it is so essential to preserve all the deciduous teeth intact to within a few months of the probable time of their being shed. In a case of normal development and where the child has no bad habits which may affect the position of any of the teeeth, I believe after the complete eruption and development of the first permanent molar it is not so absolutely essential. The temporary cuspid, however, should not be sacrificed at any price.

I will not dwell on the first permanent molar, or the cuspid, as we can with much profit to ourselves devote an entire evening to these teeth, I will now endeavor to point out some of the peculiarities which exist in the deciduous teeth and jaws after the eruption of the first permanent molar in cases of normal development and contrast that with those in the permanent teeth and jaws. The child's jaw is orthognatus or square; the permanent jaw is prognatus or protuded. The deciduous teeth set squarely in the jaw, forming a right angle. The succedanous teeth incline mesially, forming an obtuse angle. The angle of the deciduous jaw is an obtuse angle. The angle of the permanent jaw is a right angle. The pterygoid process in the child is inclined mesially forming an obtuse angle with the body of the sphenoid. The pterygoid process in the adult is perpendicular and forms a right angle; as you all know, it is between the pterygoid process and the deciduous upper jaw that the permanent upper molars erupt. The width of the deciduous arch between the temporary molars at the gum margins is said to be the same as the width between the permanent bicuspids, therefore the width of the deciduous arch in the deciduous molar region remains constant while the width of the arch increases posterior of the deciduous jaw. The length of the deciduous arch over the occluding surface is much shorter than that of the permanent arch. This is due, no doubt, to the great mesial inclination of the anterior teeth. Thus, what may seem to be a very great increase in the size of the jaw may be largely a different slant of the teeth. The sum of the mesiodistal diameters, temporary cuspid, first molar and second molar in the lower jaw is greater than that in the upper jaw. The sum of the mesiodistal diameters of the three permanent successors is less than that of the same three temporary teeth. As this sum of these three deciduous teeth is greater than the sum of the diameters of their permanent successors, and spacing has occured between all the anterior teeth and between the cuspid and first temporary molar, it must be evident that at this age in a case of normal development nature has not only created sufficient space to accommodate the incoming succedanous teeth, but even more space than is necessary, therefore, nature must also have made allowance for the mesial drifting of the first permanent molars. From this brief contrast it must be evident to you that the conditions which exist after the eruption of the first permanent molar are different than those which preceded, and that while we should never extract a deciduous tooth before complete eruption of the first permanent molar, after which time the rule does not so generally apply, with the exception of a temporary cuspid, which should never be lost permaturely.

However, granting the idea, what is the best procedure for the many cases that present themselves in daily practice?

Is there a simple procedure which will render good service in those cases in which we may not be properly remunerated or in cases where the ravages of decay have been so extensive as to make the teeth beyond repair. Is extraction ever justifiable, and, if so, is there not a favorable time? The essayist has said where the condition of a child demands removal of his deciduous teeth, that space thus produced should invariably be retained mechanically unless the permanent teeth can be expected to erupt within a few months. The essayist has admitted that the extraction of a deciduous tooth is sometimes permissible, but how about the many cases where the insertion of a retaining appliance is impossible and improper, especially where the patient has not the necessary means to remunerate the operator. Should we not, therefore, be just a little more generous in our criticisms of those operators who are so unfortunate as to be compelled to resort to such practices?

By paliative treatment many deciduous teeth can be preserved in a comfortable condition, and the extraction of many of their roots avoided. It is also well to remember that while it is important that we preserve the roots of the deciduous teeth, it is also a good idea to preserve the roots of badly broken down first permanent molars. While the essayist says that he has repeatedly seen first permanent molars tipped forward and receded as a result of premature loss of deciduous molars, I think many of us can readily recall cases of premature loss of deciduous molars in which there has been no malocclusion resulting. The question, therefore, arises, why is it that in some cases there is malocclusion and in others there is no malocclusion? What were the conditions preceding and following their premature loss? That word "premature" is very indefinite. After the age of 8 the first permanent molar is, as a rule, completely erupted, and the widest part of the incoming permanent teeth is already formed, with the exception of the third permanent molar; and as the time which elapses before the eruption of the incoming permanent teeth is of short duration, it must be evident that it is more favorable to extract a deciduous molar after this time than previous thereto, and that there will be little danger of malocclusion resulting if conditions are normal. By this it is not to be inferred that it is not necessary to preserve the deciduous molars after this age, for these teeth should always be saved whenever possible. It is to be remembered that if you mean only to refuse the extraction of a deciduous molar, that may mean that we are drifting away from the ideal, and the subject then changes to the complex, under which the production of symmetrical conditions in both jaws by extracting should be avoided. The assymmetrical conditions are always more favorable.

To thoroughly convince the laity of the importance of the deciduous teeth is not an easy task, for they have inherited an impression that these teeth are of no consequence, so that it will require a great deal more time and persuasion than the average dentist has in his possession. It is, however, true that the average dentist does not do his duty to sufficiently educate the public. If more time were devoted to instructing our patients while operating for them, as to proper protective dental service, instead of talking usually about every other subject under the sun, much more rapid advancement would result. While it is true that patients lay too much stress altogether on the correction of dental deformities, it is not necessarily a logical conclusion, as the essayist says, that patients will go to double the expense to give the deciduous teeth the best possible attention. In this city I doubt if there are more than a dozen dentists who can practice exclusively along ideal lines, and even with these there will be a time when they have not the honesty of shifting responsibility by sending away undesirable cases to other practitioners if they persist in the ideal. Is it not, therefore, just as unprofessional for such practitioners to refer such cases to offices where they will fail to receive such treatment? At least it would not be Christian like—not to criticise the other practitioner too severely.

It has been very clearly pointed out to us by the essayist, that all which can be hoped for in enlarging the deciduous jaws is to assist nature in carrying out its own plans, and that this early treatment does not necessarily insure every tooth erupting in its normal position. It must be clear, however, that the correction of a mal-posed tooth under such favorable conditions is a very simple operation when compared to the complex conditions which follow where treatment has been deferred until later on in life. We often meet with cases where a few of the anterior deciduous teeth are in slight torsal, lingual or labial occlusion, and it must be very plain that to treat such cases, if conditions are otherwise normal, would be carrying the idealism on to a degree of madness, inasmuch as we do not know where the permanent teeth will erupt.

Dr. JOHN S. HALL. The discussion of this paper that was brought out by Dr. Laupe, was done so well that it is hardly necessary for me to talk further on it. I am sure there are a goodly number of dentists who are so situated as to have patients who have so little regard for the care of their own teeth and so little knowledge of the proper care of them, that it hardly seems necessary for them to give any attention to the deciduous teeth of their children.

You all have brought to your offices children suffering pain, and you tell the parents about the necessity of having the tooth treated and saved and the good that will eventually follow from having that done, it really seems like a waste of time and talk as they will not tolerate anything short of immediate relief. I think much criticism has been brought upon many dentists in the past in this way. There is a difference with the dentists where they are located where they don't meet these conditions. You must remember that in a city of this size there are different settlements of foreigners largely; these children of foreigners eventually make American citizens and need teeth just as the Americans do, but the difficulty is to make them see that there should be anything done for them. I think if the dentists will just think about that they will sometimes be less harsh in their criticisms of dentists who sometimes are forced to extract those teeth. I have been guilty of doing that, and possibly, and likely, will be guilty of doing it again. I should like you to show me how I can get out of that, when or what I shall do. If you can show that to me in a sort of tangible, practical, workable way, you will have done me much good, and I will be able possibly to do much good to these children, but the persuasive eloquence I have has failed largely up to the present time. Possibly the seeds have been falling on stony places; they are likely to fall upon stony and rocky places for quite a while yet. If by persistence we may expect some little return in the line of reaping a harvest of good, why, we will continue; but it seems to me if any of these patients where I have extracted a tooth and have come under your criticism because of your higher ideal, from never having had any experience in that line, I ask you to be less harsh, not that I care so much about the criticism, because I feel that possibly I am doing some good work where I am, possibly just as much good as some of you fellows who are doing so much criticising. I am very glad to have this opportunity to bring news from the far outskirts of the city to tell you about conditions that happen down there.

Dr. O. W. White. Mr. President, I wish to congratulate Dr. Watson on the very able manner in which he has presented this subject to-night. This subject is one that dental and medical journals have been giving great publicity to, during the last year. During the first few years of the child's life, say from two years to six, we have so many things which affect the development of the dental arch. The teeth, process, bones and tissues which make it, and at birth probably $\frac{1}{6}$ of the face is developed. At this time, the teeth occupy all the space below the orbit and the sinus is very small. Probably the greatest influence in bringing the palate down as the face grows is by the suction of the thumb. If we had any way of measuring the pressure produced by the tongue on the palate you would easily realize that it is probably the greatest force in the distension of the maxillae.

I have been fortunate enough to treat a little patient three years of age who developed the habit of thumb-sucking, producing a protrusion of the upper of half an inch. I commenced treatment at three years of age. The child not realizing the benefits to be derived, one would expect a great deal of trouble; but the child wore the arch without trouble, and I produced a normal dental arch at five years of age. The second molars will now develop into normal occlusion. They cannot be otherwise.

I think a great deal of success can be obtained from spreading the arch in the bicuspid region between the years of four and five. One thing you can and always should do is to correct any mesial or distal occlusion of the arches, then you can expect the arches to develop normally in that position, and that will save one of the greatest difficulties which you will encounter later.

DR. C. H. LAND. I lived through the time when we had no rhinologists, and we had no orthodontists, and yet there are cases to-day that require one of these specialties. I had 11 cases one year to regulate; they presented some very remarkable features. I succeeded to a large extent, because I had compassion on the various people who came to me for services, and after sending out two cases, I took charge myself. At the time I had probably the largest practice in what we now call orthodontia of any one in the city. The result of these cases after 20 years experience was: I want to say to you that I find the majority of cases that need attention of the Orthodontist are those cases which require a very high skill to preserve and protect them after the orthodontist gets through, and the majority result in a very large failure. When I find normal conditions I found very little trouble. I find that some parents do not appreciate these things; nine times out of ten it is want of money; we want proper fees for these difficult operations.

I am glad to see the time when we have Orthodontia specialists, when we have men to work and who devote their whole time and attention to these things. I have a case now where I need an orthodontist and where I will need men skilled to accomplish a practical result. For instance,

I have one case where there is no normally developed teeth of the 28 teeth, where the orifice is so narrow that it had to be expanded for an inch. I will have to refer that to an orthodontist to expand that mouth and bring to the proper antagonism. I shall refer it to some one and let them take care of it, and in the end I will put on my porcelain crown, because I never put gold crowns on teeth like that. I have had cases like that that were saved for 15 or 20 years, and all with their pulps in yet.

Dr. C. P. Wood. I want to say that this discussion is by far the most important that I have listened to this year in this society or any other dental meeting, and I think it cannot help but stimulate each one of us to higher appreciation of the forces of orthodontia and of the proper time to refer these cases.

I think that the most important feature or part of the paper for each one of us is that which referred to the proper filling of these temporary teeth. Dr. Watson stated, I believe, that he had observed that the older or the busiest were the ones who took the most pains with the filling of the temporary teeth, and did bring about the best results. Now I am sorry to say that that has not been my experience in my own practice. I started out with the idea, when I began practice, that these temporary teeth needed contouring with a metal filling, just as thoroughly as the permanent teeth, and I am very proud and glad to say that years ago I contoured those teeth better than I do to-day, when I am a little more busy than I was then, and I am ashamed to say that I have not, for some four or five years, paid the attention to contouring the temporary molars that I did in former years. I shall, I am very sure, put more time on these cases in the future, and spend a little more nerve force in bringing that child up to the point of having that same filling, if you please. I believe the metal filling for the temporary teeth is what should be put in and contoured. Of course, I realize the difficulty of putting in the metal filling in these temporary teeth where we have not much room between the outer shell and the pulp, but I think it is highly important that we, as regular practitioners do that, and in connection with the possibilities and probabilities of future irregularities caused by the loss of space.

DR. T. R. BUTTRICK. I think Dr. Watson intimated that there are times when it is absolutely necessary to extract deciduous teeth, and this needs no discussion. I think that the ideal Dr. Watson put forth in his paper is one that none of us can take any exception to, and I think the nearer we come to that ideal the better. There is an old French proverb that he who excuses himself accuses himself. There is no question but that all of us have extracted deciduous teeth. Unfortunately the little people come to us, sometimes with the teeth so far gone, and they themselves so nervous that you cannot do what you would like to do, but there is no questioning the fact of what teeth should be saved and fillings should be inserted that will preserve the meso-distal diameters. In my own practice I try to get contour as nearly as I can, and I think amalgam is perhaps the best material that we have for children's teeth. When it comes to treating these little molars and curing abscesses where the roots are largely absorbed, wide open before the eye, I do not think any of us can do ideal work, but the nearer we come to what we know we should do, the more satisfied we ought to be with what we accomplish.

Of course there are many children that cannot afford proper dental surgery, and to such I hope our free clinics will be to them what the offices of some of the dentists here are for the public.

In Dr. Laupe's discussion I find a number of things. I listened rather attentively to the dentition of the teeth and tried to follow him. I find he made the statement that under no circumstances should the deciduous cuspid be extracted, but did not find that he gave any real reason for that. I agree with him that the deciduous cuspid should not be extracted, but will go further, that the deciduous molar should not be extracted, if it is possible to save it.

Now I do not know that I have anything further to say. It occurred to me when Dr. White was talking of removing the cause you cured the trouble. Practically, if he had cut off the stem he may have avoided the malocclusion in the upper arch.

Dr. Watson, (closing). I appreciate thoroughly the generous discussion and the courteous treatment I have received at your hands to-night. I feel that there is little occasion for me to enter into any lengthy discussion, though there is one point which I especially wish to emphasize as it seems to have been ignored, to a greater or less degree, by several of the men who have taken part in this discussion. The point is this—the title of this paper is one that makes it necessary for us to have in mind only the very highest ideals, for in no other way will it be possible for us to aid nature by relieving her of some of the handicapping influences which contribute to facial and oral deformity. If the title of the paper had been "Dental Charities" or "The Care of the Teeth of the Poor" my recommendations would naturally have been tempered to suit the conditions.

Dr. Laupe differs with me on two or three points, the chief of which is his contention that after eight years of age the deciduous molars are of little importance because the first permanent molars are completely erupted. This argument is to my mind utterly illogical, for I believe there is no time during life when proper digestion and assimilation are of more importance than during the tremendously rapid growth of childhood, and I confess to you that I cannot believe that half of an upper and half of a lower molar, for that is really what the normal relation of these teeth amounts to, are capable of masticating the food of a child at this particular period of its existence.

